# Physician factors associated with increased risk for complaints in primary care emergency services: a case – control study

**DOI:** 10.1186/s12875-020-01272-0

**Published:** 2020-09-25

**Authors:** Svein Zander Bratland, Valborg Baste, Knut Steen, Esperanza Diaz, Svein Gjelstad, Gunnar Tschudi Bondevik

**Affiliations:** 1 National Centre for Emergency Primary Health Care, NORCE Norwegian Research Centre, Kalfarveien 31, N-5018 Bergen, Norway; 2 Department of Global Public Health and Primary Care, University of Bergen, Kalfarveien 31, N-5018 Bergen, Norway; 3 Unit for Migration and Health, Norwegian Institute of Public Health, Oslo, Norway; 4 Department of General Practice, University of Oslo, Kirkeveien 166, Fredrik Holsts hus, N-0450 Oslo, Norway

**Keywords:** General practice, General practitioners, Emergency medical services, Medical errors, Medical audit, Case-control study

## Abstract

**Background:**

Patient safety incidents defined as any unintended or unexpected incident that could have or were judged to have led to patient harm, are reported as relatively common. In this study patient complaints have been used as an indicator to uncover the occurrence of patient safety incidents in primary care emergency units (PCEUs) in Norway.

**Methods:**

Ten PCEUs in major cities and rural parts of Norway participated. These units cover one third of the Norwegian population. A case-control design was applied. The case was the physician that evoked a complaint. The controls were three randomly chosen physicians from the same PCEU as the physician having evoked the complaint. The following variables regarding the physicians were chosen: gender, citizenship at, and years after authorization as physician, and specialty in general practice. The magnitude of patient contact was defined as the workload at the PCEU. The physicians’ characteristics and workload were extracted from the medical records from the fourteen-day period prior to the consultation that elicited the complaint. The rest of the variables were then obtained from the Norwegian physician position register. Logistic regression was used to estimate odds ratio for complaints both unadjusted and adjusted for the independent variables. The data were analyzed using SPSS (Version25) and STATA.

**Results:**

A total of 78 cases and 217 controls were included during 18 months (September 1st 2015 till March 1st 2017). The risk of evoking a complaint was significantly higher for physicians without specialty in general practice, and lower for those with medium low and medium high workload compared to physicians with no duty during the fourteen-day period prior to the index consultation. The limited strength of the study did not make it possible to assess any correlation between workload and the other variables (physician’s gender, seniority and citizenship at time of authorization).

**Conclusions:**

Continuous medical training and achieving the specialty in general practice were decisively associated with a reduced risk for complaints in primary care emergency services. Future research should focus on elements promoting quality of care such as continuing education, duty rosters and other structural and organizational factors.

## Background

The occurrence of medical errors in primary health care, defined as an actual or a potential serious lapse in the standard of care, has been considered preventable in more than 90% of detected cases [1]. Learning from medical errors is therefore crucial and can be based on user surveys, reporting systems for healthcare and patient complaints [1–7]. The reasons for complaining are obviously numerous. Issues concerning treatment and communication are found as the predominant reasons [5, 8]. However, patient complaints are in their nature not necessarily related to a medical error [1, 2]. Studying unintentional incidents is accordingly challenging [5–7]. In 2006, a Norwegian study of complaints against general practitioners, indicated an association between patient complaints and the gender and citizenship of the physician [9].

Patient safety incidents (PSIs) have been defined as any unintended or unexpected incident(s) that could have or were judged to have led to patient harm [7]. These incidents may include complaints from the patients, unfortunate incidents, accidental events and medical errors. This part of our study is based on patient complaints. By reviewing the medical record (e.g. clinical auditing) connected to a patient complaint, poor clinical performance may be identified [10, 11]. We have chosen patient complaints as an appropriate outset in this context.

Norway has 5.2 million inhabitants and a low population density. In 2018, there were 177 primary care emergency units (PCEUs); 75 covering one municipality and 102 covering more than one. On this multifaceted background, we did not perceive it feasible to establish a representative study sample. Because of this we used a case-control design, as patient complaints are infrequent, and the control group uncovers the denominator of the fractions of participation in PCEU. To our knowledge patient complaints have previously not been studied using a case-control design.

For general practitioners in Norway participating in out-of-hours service is an additional duty to their regular medical tasks [9, 12, 13]. There are regulations for independent participation in this kind of duty, which requires at least 30 months clinical work after authorization as physician and having had at least 40 duties at medical emergency services provided by PCEUs. There is no formal supervision involved [13]. For general practitioners participating in these services is required. Some may choose to take on more duties than scheduled. This outlines the structural and organizational factors in studying PSIs in Norway.

Out-of-hours primary care is pointed out as of particular importance to patient safety [10].

Lack of information on patients’ medical history, insufficient medical knowledge, and high workload could all play a part. Little is known about the specific challenges that have to be met by the general practitioners in the PCEUs, where quick decisions and immediate actions often are required [2, 10]. This has induced the hypotheses that communication skills and experience are important factors in preventing complaints. The spoken word in a consultation is vital. We surmise that if the use of appropriate language is poor or comprehension of the patient’s view is not ideal, problems may arise. It should be expected that training, as with increasing work experience, may reduce the risk for patient complaints. Increasing experience in terms of training and seniority as a practitioner, should reduce the occurrence of complaints. These elements are specified in the requirements for achieving the specialty in general practice (GP) [13]. We have included workload, as expressed by the extent of patient contacts, as a factor possibly associated with complaints.

### Objective

The aim of this study was to examine the associations between characteristics of physicians working in ten regular PCEUs, their workload and the risk of patient complaints. This study is the first part of a project having learning from medical errors as a principal objective.

## Methods

A case-control design was used to assess if the risk of inducing patient complaint is associated with physician factors including their workload in a PCEU setting. The case is a physician at a PCEU, evoking a complaint. The controls are three randomly chosen physicians on duty in the fourteen-day period prior to the case consultation. In this part of the project the content of the complaints has not been assessed.

### Participants

Our aim on PCEU participation, was to cover one third of the Norwegian inhabitants living in urban and rural parts. To reach this we invited ten PCEUs to participate in this project. Six of these units were serving major cities and four serving mainly rural areas. The chosen units cover approximately 1.7 million people. Based on an Irish study [2], we stipulated that about 250 complaints in total would be received during 1 year. Due to challenges of handling the customized computerized data extraction programme, we chose not to use a random sample of PCEUs. We selected the largest ones with a staffing that were expected to be able to handle the extraction programme..

To facilitate a unified approach to this project, each PCEU was visited twice given oral and written guidelines on inclusion and exclusion of cases, and the use of a customized computerized data extraction programme for encrypted transmission of data from the medical records. By this data extraction we acquired the UPIN (unique physician identification number) and the parameters on workload during the fourteen-day period prior to the index consultation.

A complaint was defined as any written utterance of discontent with the physician’s measures, sent directly to the unit or via external authorities. The customized computer programme randomly selected three control-physicians for each case-physician. The control-physicians were sourced from the same unit and had been on duty the same or a previous day as the case-physician. Consequently, a case-physician could turn up as a control for another case and vice versa.

In the case group the medical record related to the complaint was acquired by the described computer program. As controls the computer randomly selected three medical records written by the three different physicians correlating in time with the case-record. The medical records were used for information about the physician characteristics and on workload. For this the UPIN was extracted together with the history of duties with numbers of patients during the fourteen-day period prior to the index consultation.

The information extracted from the medical records, was sent encrypted from the project employee to the proprietor of the Norwegian physician position register (Legestillingsregisteret - LSR). In this way the specified data was extracted and made unidentifiable for the research group, meeting the ethical considerations (see Human subject review).

The start of data collection was delayed due to necessary adjustments in the customized computer programme. The compliance with the project guidelines on including complaints, varied with no relation between the size of the PCEU and the number of included cases. We prolonged the data collection period from 12 to 18 months. Nonetheless just one third of the anticipated total of 250 complaints was submitted from the ten included PCEUs. Three complaints did not meet the inclusion criteria having no relevance to the delivered care of the physician. The data collection started September 1st 2015 and was prolonged till March 1st 2017.

### Variables

The UPINs were sent encrypted to the LSR. From this register the following information about the physicians was extracted: gender, citizenship (Norwegian vs non-Norwegian) and age at authorization, and specialty in GP. The LSR does not provide any information on citizenship change. Seniority is described as number of years after authorization as physician.

The workload factor, defined as number of patients divided by number of duties, was grouped into five categories. The first category consisted of those having no duty during the fourteen-day period prior to the index consultation. This group was used as reference. The remaining groups were divided into quartiles defining workload: Low (1 to < 6.6 no. of patients/no. of duties), Medium low (6.6 to < 8.7), Medium high (8.7 to < 12.0) and High (12.0 and higher).

**Fig. 1:**
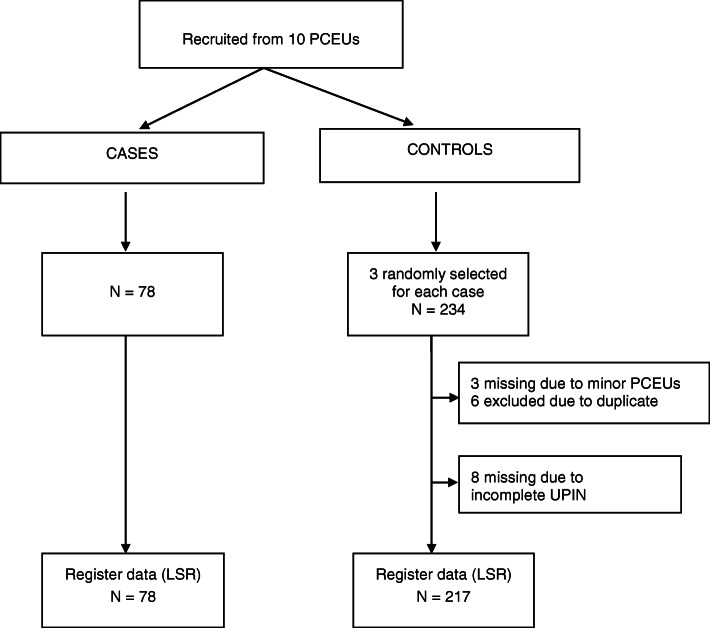
Flowchart of data in a case control study of complaints in ten Primary care emergency units (PCEUs) in Norway. Data are collected from medical records and the Norwegian physician position register (LSR) using the Unique Physician Identification Number (UPIN). PCEUs covering 1.7 mill. Inhabitants. Data collected during 18 months 2015–2017

For all cases the specified data were accessible in the medical records. For the controls 96.2% of these data were complete. The duty roster at some of the minor units, did not have three different physicians to choose from as controls for the fourteen-day period of inclusion. The rest was missing caused by incompatibility problems processing data from different systems for electronic medical records used by the participating units. The extractions from LSR reduced the complete data sets in the control group to 92.7%. This was mainly due to unidentifiable UPIN (Fig. 1).

### Statistical analyses

Descriptive statistics are presented as percentages, mean values and standard deviation (SD). Differences in mean values were tested by independent t-test. Logistic regression was used to estimate odds ratios (OR) with 95% confidence interval (CI), as a measure of association between a given characteristic related to the patient complaints (case group) compared with no complaints (controls). Multivariable logistic regression was performed to adjust for the independent variables in the same model: gender, years after authorization as physician, citizenship at authorization, specialty in GP, and workload. Robust standard error estimates were used in the regression to account for cluster of complaints within physicians. Interaction between workload and the physician’s gender, seniority, citizenship, specialty in GP, were analyzed in separate models. In addition, sensitivity analyses were performed by including physicians that had only one consultation. The data were analyzed using SPSS (Version25) and STATA 15, level of significance was set to α = 0.05.

### Human subjects review

The data collection was subjugated to ethical considerations, and consent was obtained to retrieve personal sensitive information from the medical records (2013/99/REK vest). The approval made the UPIN accessible and thereby the parameters on workload obtained from the medical records. Based on this approval, the register data in the LSR were made available by the proprietor. All transmission of information was encrypted using Secure File Transfer Protocol (SFTP).

## Results

In total, 78 cases and 217 controls (Fig. 1) involving 203 physicians (54 in case and 149 in control group, 82 females, 121 males), were included. Eight physicians (10.3%) had evoked more than one complaint. The mean age in the case group was 40.6 years and 41.4 years in the control group. Number of years post authorization as physician was 7.8 among the cases and 9.6 in the control group. Mean age at the time of the incident and the mean number of years post authorization were not significantly different for the case and control groups (*p* = 0.56 and *p* = 0.11).

Mean number of duties during the fourteen-day period of registration were 4.0 in the case group and 5.0 in the control group (*p* = 0.17). The mean total numbers of patients were 39.7 and 43.5 in case and control groups (*p* = 0.55). The highest number of patients during the 2 weeks was 215, and 27 for duties. The lowest numbers were one for both variables.

Table 1 shows the association of the various physician characteristics and workload, with the risk of evoking patient complaints. The shown gender difference in participation, has been adjusted for. The proportion of physicians with GP specialty was 26.9% in the case group, and 42.3% in the control group. Adjusted for the other variables, physicians without GP specialty had a higher (double) risk of a complaint compared to those with a GP specialty the odds ratio (OR) was 2.05, 95% confidence interval (CI): [1.10–3.81].

**Table 1:** Characteristics of physicians and their workloads in a case-control study of 78 complaints evoked by physicians in ten Norwegian primary care units handling emergency situations, and odds ratio (OR) of complaints by physician’s characteristics and workload. Units covering 1.7 mill. Inhabitants. Data collected during 18 months 2015–2017

Variables	Complaints		No-complaints					
	Cases		Controls		Unadjusted		Adjusted^a^	
	n	(%)	n	(%)	OR	95% CI	OR	95% CI
Gender								
Female	27	(34.6)	86	(39.6)	1.0		1.0	
Male	51	(65.4)	131	(60.4)	1.24	0.72–2.14	1.45	0.81–2.61
Years since authorization ^b^					0.97	0.93–1.01	0.98	0.94–1.03
Citizenship at authorization								
Norwegian	51	(65.4)	165	(76.0)	1.0		1.0	
Non-Norwegian	27	(34.6)	52	(24.0)	1.68	0.96–2.94	1.67	0.92–3.03
Specialty general practice								
Yes	21	(26.9)	92	(42.3)	1.0		1.0	
No	57	(73.1)	125	(57.6)	**2.00**	**1.13–3.54**	**2.05**	**1.10–3.81**
Workload^c^								
No duty before index consultation	29	(37.2)	42	(19.4)	1.0			
Low	13	(16.7)	43	(19.8)	0.44	0.19–1.01	**0.33**	**0.14–0.82**
Medium low	10	(12.8)	47	(21.7)	**0.31**	**0.13–0.75**	**0.23**	**0.10–0.57**
Medium high	9	(11.5)	47	(21.7)	**0.28**	**0.11–0.72**	**0.23**	**0.08–0.65**
High	17	(21.8)	38	(17.5)	0.65	0.31–1.36	0.60	0.28–1.29

Robust standard error estimates

^a^All variables included in the model

^b^ By steps of 10 years

^c^First column the no. for just one duty in the fourteen-day period. The following four rows has the quartiles of no. of patients/no. of duties:

1.0–6.6; 6.6–8.7; 8.7–12.0 and > 12.0

In the case group 34.6% of the medical records were written by physicians with non-Norwegian citizenship at the time of authorization. In the control group this percentage was 24.0. Physicians without Norwegian citizenship had an adjusted OR of 1.67, 95% CI: [0.92–3.03] evoking a complaint compared to those with Norwegian citizenship. Similarly, no significant association was shown between neither physician’s gender nor increasing seniority, in evoking a complaint. Seniority is expressed as number of years after authorization as physician, expressed by 10 years intervals.

The distribution of workload in groups expressed by number of patients divided by number of duties, is shown in Table 1. In the case group 37.2% of the physicians and 19.4% in the control group, had no duty during the fourteen-day period prior to the index consultation. Compared to this group with no duty, the adjusted OR were significantly lower for evoking a complaint in the low, medium low and medium high workload. This group had the highest odds ratio for a complaint compared to groups with higher workload. The adjusted OR were significantly lower for evoking a complaint in the low, medium low and medium high workload, compared to no duty in the fourteen-day period. No interaction was discovered between workload and the variables gender, citizenship and specialty in GP. Sensitivity analyses restricted to physicians with only one patient in the inclusion period, gave similar results.

## Discussion

In this study from ten PCEUs in Norway of complaint evoking, we found that having a specialty in GP, or having high workload at the PCEU, was significantly associated with a reduced risk of evoking a complaint. It was shown that physicians being exposed to frequent duties, i.e. high workload, or having obtained the GP specialty, had a significant decreased risk of evoking complaints compared to no duty prior to the index consultation and no GP specialty. Gender, seniority, and citizenship, were not associated with this risk. The essential finding of this study is the influence of the physician’s qualifications by having the GP specialty, and training expressed by having a workload of more than one duty per 14 days.

The GP specialty was established in 1985. Qualifying usually takes at least 5 years after graduation as physician. The qualification rules contain nine requirements including 2 years in a colleague-based guidance group, 4 years in GP, one-year hospital service, and defined courses. Since 2012, a course on emergency medicine has been required. The authorization as a GP specialist is given by the Norwegian directorate of health [13]. The factors that act protecting against complaints in this study, may be the patient’s perception of professional confidence in the dialogue in addition to acquired skills. To our knowledge this significant manifestation of structured continuing education has not been shown in this setting before [1, 14, 15]. The proportion of the GPs qualifying for this specialty, has been increasing the last years. This increase is especially notable amongst female GPs. The results in our study that differ from others, may mainly have been mitigated by these educational requirements and programme.

In our study, workload in the PCEU is determined by the patient contacts acting as training for the physicians. It is therefore interesting that having no duty during a period of 14 days prior to the index consultation, seems to miss the effect of training. All the other workload groups had lower odds of evoking a complaint compared to the group with only the index duty. As being on-call is about knowing the specific professional issues, and having the appropriate knowledge on facilities and co-working, this result should not be surprising. In this context it is unexpected that similar findings are not published. The effect of structured training is well known [1, 3, 15, 16]. In this line, it should not be considered unexpected that the experience caused by increasing seniority may have the protective function against complaints as shown in this study. As other studies, increasing seniority has been shown to increase the incidence of complaints or medical errors [3, 14, 17, 18]. The reason for our finding may thus be the Norwegian system requiring a post-educational program for working in a PCEU [13].

Previous studies have revealed a significant male predominance in evoking complaints [4, 14, 18]. In one study male physicians had a 40% higher rate of recurrence than their female colleagues [6]. This has been correlated to an increasing number of female physicians, working less hours than male physicians in different work and practice types [4, 14, 18, 19]. Recently, the gender difference has been stated as fundamental in a systematic review and meta-analysis [19]. The fundamental reason may be the perceived female characteristics of empathy, self-knowledge and communication skills [20–23]. In a multivariate analysis it was shown that the female physicians in Norway participated less in the PCEU, than their male colleagues [12]. As our study indicates higher risk pattern for evoking complaints with low participation in PCEU, we have no explanation for the absence of gender difference in our study.

Our study was focusing on discontent with the physician’s measures, with language skills presupposed as a decisive factor for expedient communication. Language skills and cultural competence has been shown as a prerequisite for satisfactory communication avoiding complaints [5, 8, 17]. Clinical practice patterns of immigrant physicians doing out-of-hours work in Norway, have been shown to differ modestly from that of native Norwegian physicians [24]. This study was based on immigrant status and country of origin. Physicians not having a Norwegian citizenship, may have their communication skills influenced by their mother tongue, and a diverging approach on cultural basis in communicating with patients. Our finding that complaint evoking was equal for physicians with or without Norwegian citizenship, may be explained by the Norwegian prerequisites for working in a PCEU [13]. The consequence of these regulations coincides with the results of a study including graduates from foreign versus US medical schools, showing better patient outcome with graduates from foreign schools [25]. This is explained by a rigorous approach to incorporate international medical graduates.

Our study points out the importance of continuous medical training and education. The existing qualification system for working in PCEUs in Norway, may thus have concealed the differences related to gender and citizenship.

### Strengths and limitations

The strength of this study is the case-control design with a proper control group with valid and nearly complete data sets. This is bolstering the strength of the study. The main weakness of the study is the unexpectedly low number of complaints included.

We ended up with one third of the anticipated number of complaints [2]. There were various reasons for this: compatibility problems with the customized data extraction programme and the different electronic medical record systems, changing leadership during the study period at some PCEUs together with heavy workload. The lack of electronic compatibility was the essential reason for one of the larger units. The overall outcome being lesser alertness and curbed motivation for complying with the intention of including cases. Broad scale data extraction from different electronic medical record systems, is at present still not possible. Medicolegal issues were not included in the referred study of complaints in GP consultations for patients with urgent problems [2]. As our study had solely physician behavior-issues as a criterium for exclusion, this may have been an additional reason for this low incidence of complaints. This low number of complaints and the thereby low number of physicians included, create limitations on the application of the results of this study.

One tenth of the physicians were evoking more than one complaint during the fourteen-day period of inclusion. The reason for this rather high number, may be explained by coincidences caused by the small study sample with some of the PCEUs not having three participating physicians for randomizing. From larger studies the incidence of more than one complaint, is reported as less than 10% during a two-year period and 24.5% in a ten-year period [4, 6]. As smaller units with rather few participating physicians were included, the frequency of duties increases the probability to be picked up as a control more than once. This may be a bias in this study reflected by the lower number of individual physicians than should be expected from the number of cases. This does not seem to have influenced the results.

## Conclusions

In this study from ten PCEUs in Norway of complaint evoking, we found that having a specialty in GP, or having high workload at the PCEU, was significantly associated with a reduced risk of evoking a complaint. Gender, seniority and not having Norwegian citizenship at time of authorization as physician, were not associated with the risk of evoking a complaint.

This study points to the importance of medical training and continuing education as a serious issue that could minimize risk of evoking complaints. Future research should be focusing on the elements of exposure to and the training in handling emergency situations in primary health care.

## Data Availability

The permission was granted provided de-identification or eradicating the study material within the end of 2018. De-identification of the study material was performed in due time. The datasets used during the current study are available de-identified from the corresponding author on reasonable request.

## Abbreviations

GP: General practice, general practitioner
PCEU: Primary care emergency unit
PSI: Patient safety incident
SPSS: Statistical Package for the Social Sciences
UPIN: Unique personal identification number
